# ERCP Performed Out‐of‐Working Hours Is Effective and Reliable for the Treatment of Acute Cholangitis

**DOI:** 10.1155/grp/2344299

**Published:** 2026-04-03

**Authors:** Kerem Kenarlı, Bülent Ödemiş, Dilara Turan Gökçe, Orhan Coşkun, Volkan Gökbulut, Zeki Mesut Yalin Kılıç

**Affiliations:** ^1^ Department of Gastroenterology, Ankara City Hospital, University of Health Sciences, Ankara, Turkey, akdeniz.edu.tr

**Keywords:** biliary tract, cholangitis, emergency endoscopic treatment, endoscopic retrograde cholangiopancreatography, off-hours ERCP

## Abstract

**Background:**

Acute cholangitis is a life‐threatening condition that requires prompt biliary drainage, typically achieved through endoscopic retrograde cholangiopancreatography (ERCP). However, data on outcomes of procedures performed outside of regular working hours remain limited. This study evaluates the safety and efficacy of out‐of‐working‐hours ERCP for acute cholangitis.

**Methods:**

A retrospective analysis of 83 patients who underwent out‐of‐working‐hours ERCP for acute cholangitis was conducted. Patients were categorized by cholangitis severity (mild, moderate, and severe) and procedure timing (< 12, 12–24, and 24–48 h). Outcomes assessed included procedural success, complications, and clinical results. Multivariate logistic regression was used to identify predictors of prolonged hospitalization.

**Results:**

Of the patients, 14.5% had mild, 19.3% moderate, and 66.3% severe cholangitis. Median time to procedure was shorter in severe cases (6.0 h) than in mild (13.5) and moderate (14.5) cases (*p* = 0.016). Technical success was 100%, with 91.6% receiving stents or nasobiliary drains. Complications occurred in 6%, and mortality (3.6%) was limited to severe cases. Early intervention (< 12 h) significantly reduced prolonged hospital stays (> 15 days, *p* = 0.006).

**Conclusion:**

Out‐of‐working‐hours ERCP was associated with high technical success and a low complication rate in this cohort. Out‐of‐working‐hours ERCP achieved high rates of successful biliary drainage with an acceptable safety profile, and ERCP performed within 12 h was independently associated with a lower risk of prolonged hospitalization.

## 1. Introduction

Acute cholangitis (AC) is a bacterial infection of the sterile biliary system and is mainly accompanied by biliary stasis caused by choledocholithiasis or biliary strictures [1, 2]. Morbidity and mortality rates may worsen in the case of delayed intervention to provide biliary drainage [3–6]. Developments in both endoscopic and percutaneous biliary drainage techniques along with the increased awareness in sepsis have reduced the mortality rate, which was more than 50% before the 1970s, to 2%–11% today [5–7]. Hemodynamic resuscitation and antibiotic therapy are recommended immediately following the AC diagnosis. ERCP is the primary modality for biliary drainage and allows etiology‐oriented treatment in patients with AC [3, 4].

Although most cases of mild acute cholangitis usually resolve with initial conservative treatment, early ERCP is required in patients with moderate to severe cholangitis [4, 8]. It has been shown that every 1‐day delay in performing ERCP is associated with a 17% increase in permanent organ failure [5]. However, although studies indicate reduced morbidity and mortality along with fewer costs by performing ERCP on weekends in patients with AC [9–11], there are concerns regarding the effectiveness and safety of ERCP performed out of working hours. This study is aimed at evaluating the effectiveness and reliability of ERCP performed to treat AC during out of working hours.

## 2. Materials and Methods

The current study was designed as a retrospective cohort study and conducted at a third‐level medical facility. The study was approved by the Scientific Research Assessment and Ethics Committee of the same institution (No. E2‐21‐610).

### 2.1. Study Population

All consecutive eligible patients admitted to the emergency department and diagnosed with AC at our center over a 2‐year period were retrospectively screened. Patients who underwent urgent ERCP out of working hours were screened. A total of 91 patients with AC underwent urgent ERCP out of working hours. Concomitant acute pancreatitis was considered a major confounder, as it may independently affect inflammatory markers, clinical course, intensive care unit (ICU) requirement, length of stay, and adverse events. Therefore, to evaluate outcomes attributable primarily to AC and out‐of‐working‐hours ERCP, these patients were excluded. Among the study population (*n* = 83), 22 patients, 15 in the province and 7 out of the province, were referred to our hospital from other centers for the need of urgent ERCP. Intravenous fluid resuscitation and antibiotic therapy were initiated in all patients before the ERCP procedure.

### 2.2. Definitions and Data Collection

The diagnosis and severity of AC were made according to the corresponding Tokyo Guidelines 2018 [12]. ERCP time was defined as the time between the emergency department admission and the ERCP initiation. The concept of “out‐of‐working‐hours” defined the period between 5 p.m. and 8 a.m. on weekdays in addition to the weekends and national holidays (approximately 20 days per year) when routine ERCP procedures are not carried out. Out‐of‐working‐hours ERCP was performed under the same procedural conditions as during regular working hours, including the same on‐call endoscopists, identical endoscopic equipment, and 24‐h availability of an anesthesiology team providing propofol‐based deep sedation.

Technical success was defined as the achievement of effective biliary drainage during the index ERCP, either by placement of a biliary stent or nasobiliary drain (NBD), or by complete clearance of the biliary obstruction when considered safe and feasible. In the emergency setting of AC, definitive stone extraction was not mandatory for technical success.

The Charlson comorbidity index (CCI) was calculated to evaluate the comorbidity status of the study population.

### 2.3. ERCP Procedure

Written informed consent was obtained from all patients for the endoscopic procedure. Two experienced pancreaticobiliary endoscopists performed all procedures using two models of duodenoscope (TJF 260V and JF 260V, Olympus, Tokyo, Japan).

All procedures were accompanied by an experienced ERCP nurse, a radiology technician, a last‐year gastroenterology resident, and an anesthesiologist.

All procedures were performed under propofol‐based deep sedation administered by an anesthesiology team. A dedicated on‐call anesthesiology service was available 24 h a day; therefore, the sedation strategy was identical during working hours and out of working hours. Intravenous hyoscine‐N‐butylbromide or glucagon was administered to inhibit intestinal peristalsis during the procedure, if necessary. A standard wire‐guided biliary cannulation method was used for biliary cannulation in all patients. In patients with naive papillae who failed selective biliary cannulation with the standard method, rescue methods such as needle knife fistulotomy, double guidewire cannulation, or guidewire‐orientation/precut following pancreatic stenting were employed. Precut was performed by using the electrosurgical system in “*force-coag-2*” mode (ESG‐100, Olympus, Japan) to avoid bleeding in patients with a high risk of bleeding due to an increased INR value (> 1.5) or antithrombotic medication use other than acetylsalicylic acid. Coagulation parameters were assessed before the procedure. In patients with significant coagulopathy, endoscopic sphincterotomy (EST) was avoided and biliary drainage was achieved by stent or NBD placement to ensure urgent source control.

Following biliary cannulation, a cholangiogram was obtained to demonstrate the cholangitis etiology. The endoscopist decided on EST, considering the patient′s coagulation parameters, anticoagulant use, etiology, and severity of cholangitis. If necessary, endoscopic papillary large balloon dilation (EPLBD) was performed with a 12‐mm diameter balloon. Common bile duct stones were removed using an extraction balloon or dormia basket. Biliary drainage was secured with plastic and/or metallic stents or an NBD catheter when necessary. A prophylactic pancreatic stent was placed for pancreatitis prophylaxis in patients with repeated insertion of the guidewire into the pancreatic duct during cannulation.

### 2.4. Statistical Analysis

Statistical analysis was performed to compare groups, evaluate associations between variables, and identify factors affecting prolonged hospitalization. NCSS (Number Cruncher Statistical System) 2007 (Kaysville, Utah, United States) program was used for statistical analysis. Continuous variables were expressed as mean ± standard deviation (SD) or median (min–max), and categorical variables as number and percentage. The suitability of quantitative data to normal distribution was tested by the Kolmogorov–Smirnov, Shapiro–Wilk test, and graphical evaluations. Mann–Whitney *U* test was used for comparing variables that did not show the normal distribution in the two groups. Kruskal–Wallis test was used for comparisons of three or more groups that did not show normal distribution, and the Bonferroni–Dunn test was used for paired comparisons. In comparing qualitative data, the Pearson chi‐square test, Fisher–Freeman–Halton exact test, and Fisher′s exact test were used. Spearman′s correlation analysis was used to evaluate the relationship between quantitative variables. Variables affecting the length of hospital stay were evaluated using logistic regression analysis. Variables with *p* < 0.10 in univariate analysis and clinically relevant parameters (age, CCI, and cholangitis severity) were entered into the multivariate logistic regression model to identify independent predictors of prolonged hospitalization. Significance was evaluated at least at the *p* < 0.05 level.

## 3. Results

### 3.1. Demographic Data and Clinical Characteristics

A total of 83 patients diagnosed with AC were recruited for the study after excluding eight patients with concomitant acute pancreatitis. The mean age (± SD) was 67.2 ± 15.0 years ranging from 27 to 90 years. Although 60.2% of the patients were under 75 years old, 39.8% were 75 years old and above, and 43 (51.8%) were male.

Thirty patients (36.2%) had no comorbid diseases. Ischemic heart disease was the most common comorbidity and was observed in 15.7% of the patients, followed by diabetes mellitus (13.3%), hypertension (10.8%), and chronic obstructive pulmonary disease (9.6%).

Majority (*n* = 61, 73.5%) of the patients did not use any anticoagulant or antiaggregant drugs. Twelve (14.5%) patients used acetylsalicylic acid. The remaining 10 patients were on antiaggregant/coagulant drugs such as clopidogrel, warfarin, or dabigatran, which significantly increase the risk of bleeding during EST.

The study group was distributed according to the severity of cholangitis: Severe cholangitis in 55 (66.3%), moderate cholangitis in 16 (19.3%), and mild cholangitis in 12 (14.5%) patients. Biliary pathology leading to cholangitis was choledocholithiasis in the vast majority of patients (*n* = 61, 73.5%), followed by malignant biliary stricture (MBS) (*n* = 12, 14.5%) and benign biliary stricture (BBS) (*n* = 10, 12.0%).

Although the major papilla was naive in 45 (54.2%) patients, the remaining 38 (45.8%) patients had a previous history of EST with (*n* = 12, 14.5%) or without (*n* = 26, 31.3%) biliary stent. The demographic and clinical characteristics of the patients are summarized in Table 1.

**Table 1:** The demographic and clinical characteristics of the patients.

		Study population (*n* = 83)	
		*n*	%
Age (years)^a^	< 75 years old	50	60.2
	≥ 75 years old	33	39.8
Gender	Male	43	51.8
	Female	40	48.2
Comorbid diseases	None	30	36.2
	Ischemic heart disease	13	15.7
	Diabetes mellitus	11	13.3
	Hypertension	9	10.8
	COPD	8	9.6
	Other	7	8.4
	Multiple	5	6.0
Antiaggregant and anticoagulant status	None	61	73.5
	Acetyl salicylic acid	12	14.5
	Clopidogrel	6	7.2
	Warfarin	2	2.4
	Dabigatran	1	1.2
	Acetylsalicylic acid + clopidogrel	1	1.2
Cholangitis severity	Mild	12	14.5
	Moderate	16	19.3
	Severe	55	66.3
Etiology	Choledocholithiasis	61	73.5
	BBS	10	12.0
	MBS	12	14.5
Papilla status	Naive	45	54.2
	EST	26	31.3
	EST + biliary stent	12	14.5

Abbreviations: BBS, benign biliary stricture; COPD, chronic obstructive pulmonary disease; EST, endoscopic sphincterotomy; MBS, malignant biliary stricture.

^a^Mean age was 67.2 ± 15.0 years.

### 3.2. Technical Details of the ERCP Procedures

All patients underwent ERCP under propofol‐based deep sedation with anesthesiology support. Selective biliary cannulation was achieved in all patients at the first ERCP session. In 45 patients with naive papillae, biliary cannulation was achieved with standard methods in 38 (84.4%) patients, needle knife fistulotomy in four (8.9%) patients, anterior incision over the pancreatic stent in two (4.4%) patients, and the double guidewire method in one (2.2%) patient (Table 2). Serum INR level was high in only one patient who underwent fistulotomy (INR: 1.89). Fistulotomy was performed using *“force-coag-2”* flow (ESG‐100 Electrosurgical Systems, Olympus, Japan) in this patient who had severe cholangitis. Only EST was applied to 20 (44.4%) of 45 patients with a naive papilla, and EST + EPLBD was applied to two (4.4%) patients (Table 2). Both patients with EST + EPLBD had no coagulopathy and no antithrombotic drug use and had moderate cholangitis due to choledocholithiasis. The stones were cleared entirely in both patients following 12‐mm EPLBD, and biliary stenting was not required. In 23 (51.1%) of 45 patients with naive papillae, EST was contraindicated, and a biliary stent, NBD, or both were placed without EST.

**Table 2:** ERCP procedure‐related findings.

		*n*	%
Cannulation method in naive papillae (*n* = 45)	Standard	38	84.4
	Fistulotomy	4	8.9
	Anterior incision over the pancreatic stent	2	4.4
	Double guidewire	1	2.2
Procedural success	Yes	83	100.0
EST in naive papillae (*n* = 45)	No	23	51.1
	Yes	20	44.4
	EST + EPLBD	2	4.4
Placing material in CBD after ERCP	No	7	8.4
	Plastic stent	58	69.9
	Metallic stent	1	1.2
	NBD	7	8.4
	Stent + NBD	10	12.0

Abbreviations: CBD, common bile duct; EPLBD, endoscopic papillary large balloon dilatation; ERCP, endoscopic retrograde cholangiopancreatography; EST, endoscopic sphincterotomy; NBD, nasobiliary drain.

In seven (11.4%) of 61 patients with choledocholithiasis, stones were extracted totally in the first procedure, and no stent was required. Two of these patients had mild cholangitis with naive papilla, three had moderate cholangitis (two patients with naive papilla and one patient with sphincterotomy), and two had severe cholangitis (one patient with naive papilla and one patient with sphincterotomy). In the remaining 54 patients with choledocholithiasis, plastic stents were placed in 43 patients, plastic stent + NBD was applied to six patients, and NBD was applied to five patients. NBD dislodgment occurred in one patient, requiring a repeat ERCP, and was considered a procedure‐related adverse event. A 5‐F plastic pancreatic stent was placed for post‐ERCP pancreatitis prophylaxis in five patients with choledocholithiasis.

A plastic biliary stent was placed in 8 of 10 patients with BBS as the etiology of cholangitis. Although NBD was placed in addition to the biliary stent in one of the remaining patients, the other patient was followed up with only NBD. In the case of 12 patients with MBS, biliary drainage was constituted with plastic biliary stents in seven patients. Among the remaining five patients, three had NBD in addition to a plastic biliary stent, one had NBD solely, and one had a metallic biliary stent. Technical details of the ERCP procedure are shown in Table 2. Stent + NBD application was more frequent than stent application alone in severe cholangitis compared with mild and moderate cholangitis (*p* = 0.038).

### 3.3. Post‐ERCP Complications

ERCP‐related complications were seen in five (6.0%) patients, including bleeding in four (4.8%) patients and post‐ERCP pancreatitis in one (1.2%). Of the four patients with post‐ERCP bleeding complications, only EST was applied to three patients, and EST + EPLBD was applied to one patient. Concerning bleeding rates according to the papillary intervention, only 3 (15.0%) of 20 patients who underwent sphincterotomy and one (50.0%) of the two patients who underwent EST + EPLBD had bleeding complications. Bleeding was self‐limited in three of four patients; one patient required a papillary epinephrine injection. However, endoscopic evaluation was repeated due to the presence of melena and a decrease in hemoglobin in these four patients. Bleeding had been stopped spontaneously in one patient. The remaining three patients required intervention with a heater probe following a submucosal epinephrine injection, and a biliary fully covered self‐expandable metallic stents (FCSEMS) were placed along with 7‐F NBD for biliary irrigation. Bleeding was stopped endoscopically in all three patients. Post‐ERCP pancreatitis was observed in only one patient as mild pancreatitis. The patient had moderate cholangitis and naive papillae. Biliary cannulation was achieved with the standard method without sphincterotomy application. No other complication, such as perforation, occurred in the study population.

### 3.4. Evaluation of Patients According to Cholangitis Severity

The median ERCP time was 8.5 h in the study population, ranging from 1 to 44 h. The median ERCP time was significantly shorter in patients with severe cholangitis (6.0 h) than in moderate (14.5 h) and mild cholangitis (13.5 h) groups (*p* = 0.016).

In 43.4% of the cases (*n* = 36), papillary pus was observed during ERCP, and there was no significant correlation between cholangitis stage and presence of the papillary pus (*p* = 0.992).

The median duration of hospitalization was 8.0, 11.5, and 12.0 days in mild, moderate, and severe cholangitis groups, respectively (*p* = 0.384). There was no statistically significant difference in the need for ICU among the cholangitis severity groups (*p* = 0.092).

The median values of CCI were 1.5, 3.5, and 4.0 in mild, moderate, and severe cholangitis groups, respectively (*p* = 0.028). Pairwise comparison between mild and moderate, mild and severe, and moderate and severe cholangitis revealed *p* values of 0.120, 0.008, and 0.375, respectively.

Mortality was observed in only three patients, all in the severe cholangitis group. Of the four patients with bleeding, two patients had mild, one had moderate, and one had severe cholangitis. There was no statistically significant difference between cholangitis severity groups in terms of bleeding frequency (*p* = 0.068). Patients were further evaluated according to cholangitis severity, as summarized in Table 3.

**Table 3:** The evaluation of patients according to cholangitis severity.

	Total (*n* = 83)	Cholangitis severity			*p*value
		Mild (*n* = 12)	Moderate (*n* = 16)	Severe (*n* = 55)	
Time to ERCP (hours), median (min–max)	8.5 (1.0–44.0)	13.5 (1.5–44.0)	14.5 (1.5–41.0)	6.0 (1.0–30.0)	0.016^a^
Purulent bile on papilla, *n* (%)	36 (43.4)	5 (41.7)	7 (43.8)	24 (43.6)	0.992^b^
Duration of hospitalization (days), median (min–max)	11.0 (1.0–87.0)	8.0 (2.0–35.0)	11.5 (4.0–46.0)	12.0 (1.0–87.0)	0.384^a^
Hospitalization in ICU, *n* (%)	45 (54.2)	6 (50.0)	5 (31.2)	34 (61.8)	0.092^b^
Charlson comorbidity index, median (min–max)	4.0 (0.0–10.0)	1.5 (0.0–7.0)	3.5 (0.0–9.0)	4.0 (0.0–10.0)	0.028
Mortality, *n* (%)	3 (3.6)	0 (0)	0 (0)	3 (5.5)	N/A
Complications, *n* (%)					
No Bleeding Post‐ERCP pancreatitis	78 (94.0)	10 (83.3)	14 (87.5)	54 (98.2)	0.039
	4 (4.8)	2 (16.7)	1 (6.3)	1 (1.8)	0.068
	1 (1.2)	0 (0.0)	1 (6.3)	0 (0)	N/A

Abbreviations: ERCP, endoscopic retrograde cholangiopancreatography; ICU, intensive care unit; N/A, not applicable.

^a^Kruskal Wallis test.

^b^Pearson chi‐square test.

^c^Fisher–Freeman–Halton exact test.

Laboratory findings were evaluated according to the severity of cholangitis (Table S1). Transaminase and cholestatic enzyme levels were the same among severity groups. Serum creatinine was significantly higher in patients with severe cholangitis (*p* < 0.001). Although leukocyte and neutrophil counts were higher in patients with moderate and severe cholangitis than in the mild group (*p* = 0.001 and *p* = 0.002, respectively), the platelet count was lower in severe cholangitis compared with others (*p* < 0.001). INR and CRP levels were significantly higher in severe cholangitis (*p* < 0.001 and *p* = 0.001, respectively).

### 3.5. Evaluation of the Patients According to Procedural Timing of ERCP

The association of different ERCP timing periods with cholangitis severity and the impact of timing of ERCP on the need for ICU admission and mortality were given in Table 4. The rate of performing ERCP in the first 12 h was higher in patients with severe cholangitis than in the mild and moderate groups (*p* = 0.041). There was no significant difference in ERCP timing between mild and moderate cholangitis cases (*p* > 0.05). ERCP timing was the same in patients who required ICU admission and those who did not (*p* = 0.998).

**Table 4:** Evaluation of the patients according to procedural timing of ERCP.

		ERCP time			*p*valuea
		< 12 h (*n* = 51)	12–24 h (*n* = 24)	24–48 h (*n* = 8)	
Cholangitis severity, *n* (%)	Mild	5 (9.8)	5 (20.8)	2 (25.0)	0.041
	Moderate	7 (13.7)	6 (25.0)	3 (37.5)	
	Severe	39 (76.5)	13 (54.2)	3 (37.5)	
ICU hospitalization, *n* (%)	No	21 (45.7)	12 (44.4)	5 (50.0)	0.998
	Yes	25 (54.3)	15 (55.6)	5 (50.0)	
Mortality, *n* (%)	No	50 (98.0)	22 (91.7)	8 (100.0)	0.441
	Yes	1 (2.0)	2 (8.3)	0 (0.0)	

Abbreviations: ERCP, endoscopic retrograde cholangiopancreatography; ICU, intensive care unit.

^a^Fisher–Freeman–Halton exact test.

Despite adequate biliary drainage after ERCP, mortality occurred secondary to cholangitis in three (3.6%) patients with severe cholangitis. ERCP was performed within 12 h of hospital admission in one patient, and between 12 and 24 h in the remaining two patients. Because of the small number of deaths, the study was not powered to detect a significant association between ERCP timing and mortality.

The multivariate model included ERCP timing, age, CCI, and cholangitis severity. Multivariate logistic regression analysis was performed to identify independent factors associated with prolonged hospitalization. ERCP performed within the first 12 h of hospital admission was not related to ICU stay but reduced the risk of hospitalization longer than 15 days (*p* = 0.006, OR: 0.12, CI [0.027–0.542]). The 12–24‐h group was related to neither ICU stay nor the risk of hospitalization longer than 15 days.

## 4. Discussion

AC is a critical gastrointestinal emergency with high morbidity and mortality rates [13]. ERCP is the preferred method for biliary drainage in AC. Although performing urgent ERCP on weekends has shown decreased morbidity and mortality [9–11], concerns about technical success and procedural safety persist. This study of 83 patients undergoing urgent ERCP out of working hours demonstrated a high technical success rate (100%) and low complication rate (6.0%), attributable to experienced endoscopists and staff.

Postdrainage mortality in AC is approximately 10% regardless of severity [14]. Previous studies have shown that early ERCP may reduce mortality [15]. Khamaysi et al. found lower 30‐day mortality (15%) in severe AC patients undergoing urgent ERCP within 12 h compared with delayed ERCP (21%) [16]. Similarly, delayed ERCP (> 3 days) increased morbidity and mortality [17]. In this study, the overall mortality rate was 3.6% (3/83), rising to 5.5% (3/55) in severe AC. ERCP was performed within 12 h in 70% and within 24 h in 94.5% of severe AC patients. Performing ERCP within 48 h reduced mortality across all AC stages.

Studies suggest that not delaying ERCP until weekdays shortens hospital stays and is cost‐effective [18, 19]. A meta‐analysis reported a 3‐day reduction in hospital stay for ERCP performed within 24 h compared with later [19]. In this study, ERCP within 12 h significantly reduced the risk of hospital stays exceeding 15 days (OR: 0.12, *p* = 0.006). Median hospital stays in mild, moderate, and severe AC were 8.0, 11.5, and 12.0 days, respectively, with an overall median of 11.0 days. Although ERCP performed within the first 12 h was identified as an independent factor associated with a lower risk of prolonged hospitalization, this finding should be interpreted with caution. Due to the single‐arm design and the lack of a control group not undergoing early ERCP, a causal relationship cannot be established. In addition, the length of hospital stay may be influenced by comorbidities and nonbiliary clinical conditions, which may explain occasional prolonged hospitalization even in patients with mild cholangitis. Therefore, our results reflect an association rather than a definitive effect of ERCP timing. Future comparative studies including patients managed with delayed ERCP are required to confirm this observation.

For mild and moderate AC, early ERCP positively impacts prognosis. Jang et al. found no significant differences in technical or clinical success and complications between urgent and elective ERCP [8]. However, 15% of mild‐to‐moderate AC cases progressed to sepsis, emphasizing the importance of urgent decompression. In this study, 12 (14.5%) mild and 16 (19.3%) moderate AC patients underwent early ERCP with no observed disease progression, supporting early ERCP as recommended in the Tokyo Guidelines 2018 [12, 20]. Although some patients were classified as having mild cholangitis, all had imaging‐proven biliary obstruction together with biochemical cholestasis requiring definitive biliary drainage. Therefore, the decision for early ERCP was driven by the presence of an obstructive etiology rather than severity grading alone. In real‐life emergency practice, early source control is often preferred once the diagnosis is established and the endoscopy team is available in order to prevent clinical deterioration and avoid delayed intervention. This approach should not be interpreted as overtreatment but as early definitive management of obstructive cholangitis.

The high proportion of patients with severe cholangitis in our cohort is mainly explained by the referral pattern of our center, which functions as a regional emergency endoscopy center. Many patients were transferred from other hospitals after initial evaluation, resulting in a more advanced clinical presentation. In addition, out‐of‐working‐hours ERCP is more frequently required in patients with severe diseases who cannot be managed conservatively. Therefore, our cohort represents a real‐life emergency case‐mix rather than the full clinical spectrum of AC. This may limit the generalizability of our findings to centers with a lower proportion of severe cases.

In this study, the primary goal of urgent ERCP was rapid biliary decompression rather than definitive stone clearance. In patients with AC, particularly those with sepsis or coagulation disturbances, a staged approach is recommended, with initial drainage followed by elective stone extraction after clinical stabilization. Therefore, the relatively low rate of complete stone removal in the index procedure reflects a deliberate risk‐adapted strategy rather than technical failure. In this context, technical success was defined as successful biliary drainage, which was achieved in all patients. Moreover, early decompression was associated with a lower risk of prolonged hospitalization, which may balance the need for additional procedures in terms of overall resource utilization.

ERCP is technically challenging, with potential complications. Failed ERCP increases hospital stays and mortality [2, 5]. Inexperienced endoscopists or unprepared conditions heighten risks [9]. This study was conducted in our center, with ERCP performed by two experienced endoscopists (> 500 procedures/year) and a skilled support team. Technical success was 100%, with no alternative drainage methods needed. The availability of a dedicated anesthesiology team and the use of propofol‐based deep sedation in all procedures, even during out of working hours, may have contributed to high technical success and low complication rates.

When laboratory parameters were evaluated according to disease severity, leukocyte and neutrophil counts were higher in moderate cholangitis compared with severe cases. This finding may reflect sepsis‐related immune dysregulation in severe disease, where bone marrow suppression and increased peripheral consumption may lead to a blunted leukocytic response despite more advanced clinical status. In addition, the higher comorbidity burden observed in the severe group may further contribute to an impaired inflammatory response. This interpretation is supported by the significantly higher creatinine and CRP levels and lower platelet counts in patients with severe cholangitis.

The most frequent complication was bleeding [14, 21]. In this study, five (6%) patients developed procedure‐related complications, including four (4.8%) with bleeding and one (1.2%) with mild pancreatitis. The post‐EST bleeding rate (15%) was higher than the historical rate of our center (1.37%), which is likely multifactorial. Acute cholangitis is commonly associated with sepsis‐related coagulopathy, leading to elevated INR levels, thrombocytopenia, and impaired platelet function. In our cohort, patients with severe cholangitis had significantly higher INR values and lower platelet counts, both of which are well‐established risk factors for post‐sphincterotomy bleeding. Moreover, the relatively small number of patients undergoing EST may have resulted in an apparently higher bleeding rate when expressed as a percentage. Importantly, all bleeding episodes were successfully managed endoscopically, and no surgery or angiographic intervention was required, indicating that endoscopic hemostasis remains effective even in this high‐risk clinical setting. In patients with impaired coagulation, sphincterotomy was intentionally avoided and drainage was achieved without cutting the papilla, reflecting a risk‐adapted strategy prioritizing urgent biliary decompression. [22]

Although NBD provides effective temporary biliary decompression, it may be associated with tube dislodgment and patient discomfort. In our cohort, one patient required repeat ERCP due to NBD dislodgment, highlighting this method as a potential drawback of temporary drainage strategies.

### 4.1. Limitations

This study has several limitations. First, the absence of a comparator group, such as patients undergoing ERCP during working hours or those managed with delayed intervention, precludes any causal or comparative interpretation; therefore, the results should be considered descriptive outcome data from a single‐arm cohort. Second, this was a single‐center study conducted in a third‐level referral hospital with a high proportion of transferred and clinically complex patients, which resulted in a predominance of severe cholangitis and may limit the generalizability of the findings to centers with a different case‐mix. Third, patients with concomitant acute pancreatitis were excluded to obtain a more homogeneous cohort and to evaluate outcomes primarily driven by acute cholangitis; however, this approach further restricts extrapolation to mixed populations. In addition, the relatively small number of patients with benign and malignant strictures limited meaningful etiology‐based subgroup analyses. Because of the low number of mortality and ICU events, multivariate analysis for these outcomes was not performed, and these results should be interpreted descriptively. Finally, the length of hospital stay represents a multifactorial endpoint influenced by comorbidities and nonbiliary clinical conditions, which may explain occasional prolonged hospitalization even in patients with mild cholangitis.

## 5. Conclusion

ERCP performed during out of working hours by an experienced team was associated with high technical success and a low complication rate in patients with AC. Early biliary drainage was associated with a shorter hospital stay. This study demonstrates that a standardized 24/7 ERCP service with dedicated anesthesia support can be safely implemented in a third‐level medical facility.

## Author Contributions

The study′s conception and design involved participation from all authors. B.Ö. wrote the manuscript′s first draft, whereas all of the other contributors provided comments on the prior versions. The final manuscript was reviewed by each author. B.Ö.: investigation, data collection, writing—original draft preparation, reviewing and editing, and visualization. K.K.: conception and design, data acquisition, interpretation of the data, and drafting of the article. D.T.G.: conception and design, data acquisition, statistical analysis, and interpretation of the data. O.C.: methodology, investigation, and supervision. V.G.: writing—original draft preparation and reviewing and editing. Z.M.Y.K.: investigation, supervision, and writing—reviewing and editing.

## Funding

No funding was received for this manuscript.

## Disclosure

The final manuscript was approved by each author.

## Conflicts of Interest

The authors declare no conflicts of interest.

## Supporting information


**Supporting Information** Additional supporting information can be found online in the Supporting Information section. Table S1: Laboratory parameters according to cholangitis severity.

## Data Availability

The data underlying this article will be shared on reasonable request from the corresponding author.
