# Supplementary material for: ERCP Performed Out‐of‐Working Hours Is Effective and Reliable for the Treatment of Acute Cholangitis
Source: Gastroenterol Res Pract. 2026 Apr 3;2026:2344299. doi: 10.1155/grp/2344299 (PMC13051852; doi:10.1155/grp/2344299)
Supplement: Supplementary file 1 — Supporting Information Additional supporting information can be found online in the Supporting Information section. Table S1: Laboratory parameters according to cholangitis severity. [file GRP-2026-2344299-s001.docx]

**Supplementary Table 1. Laboratory parameters according to cholangitis severity**

|  | |  | **Cholangitis Severity** | | | ***^a^p value*** |
| --- | --- | --- | --- | --- | --- | --- |
|  | |  | **Mild** | **Moderate** | **Severe** |  |
| **AST (IU/L)** | | *Median (Min-Max)* | 92.5 (19-205) | 112 (58-654) | 89 (21-609) | *0.104* |
| **ALT (IU/L)** | | *Median (Min-Max)* | 175.5 (34-393) | 134.5 (16-463) | 109 (38-816) | *0.740* |
| **ALP (IU/L)** | | *Median (Min-Max)* | 241.5 (86-529) | 293.5 (19-2089) | 236 (67-923) | *0.217* |
| **GGT (IU/L)** | | *Median (Min-Max)* | 282.5 (88-939) | 414 (62-1266) | 329 (97-1347) | *0.124* |
| **Total Bilirubin (mg/dl)** | *Median (Min-Max)* | | 4.2 (0.92-10.58) | 6 (3.09-25.48) | 6.2 (0.84-24.15) | *0.111* |
| **Serum creatinine (mg/dl)** | *Median (Min-Max)* | | 0.9 (0.6-1.48) | 0.9 (0.56-1.98) | 1.6 (0.42-7.74) | *<0.001* |
| **WBC count (cells/mm^3^)** | | *Mean ± SD* | 8952 ± 3087 | 17939.4 ± 8074.2 | 16507.8 ± 8423.0 | *0.001* |
| **Neutrophil count (cells/mm^3^)** | | *Mean ± SD* | 7861.7 ± 3969.9 | 16210.0 ± 7617.6 | 14377.3 ± 7940.2 | *0.002* |
| **Platelet count (x1000)** | | *Mean ± SD* | 195.8 ± 85.0 | 240.9 ± 85.7 | 135.1 ± 80.7 | *<0.001* |
| **Albumin (g/dl)** | | *Mean ± SD* | 3.7 ± 0.6 | 3.3 ± 0.6 | 3.1 ± 0.5 | *0.004* |
| **INR** | | *Mean ± SD* | 1.2 ± 0.1 | 1.2 ± 0.1 | 1.7 ± 1.0 | *<0.001* |
| **CRP (mg/dl)** | | *Mean ± SD* | 170.3 ± 125.0 | 130.4 ± 70.9 | 206.7 ± 71.6 | *0.001* |

ALP: Alkalene Phosphatase, ALT: Alanine Aminotransferase, AST: Aspartate Aminotransferase, CRP: C-Reactive Protein, GGT: Gamma Glutamyl Transferase, INR: International Normalized Ratio, WBC: White Blood Cell

*^a^Kruskal Wallis Test*
